# Dlk/ZIP kinase-induced apoptosis in human medulloblastoma cells: requirement of the mitochondrial apoptosis pathway

**DOI:** 10.1054/bjoc.2001.2158

**Published:** 2001-11

**Authors:** D Kögel, C Reimertz, P Mech, M Poppe, M C Frühwald, H Engemann, K H Scheidtmann, J H M Prehn

**Affiliations:** Interdisciplinary Center for Clinical Research (IZKF), Research Group ‘Apoptosis and Cell Death’; Department of Pharmacology and Toxicology, Department of Pediatric Hematology and Oncology, Faculty of Medicine, Westphalian Wilhelms-University, Münster, D-48149; Institute of Genetics, University of Bonn, Bonn, D-53117, Germany

**Keywords:** Dlk/ZIP kinase, DAP kinase family, apoptosis, mitochondria, Bcl-2 family, medulloblastoma

## Abstract

Dlk/ZIP kinase is a member of the Death Associated Protein (DAP) kinase family of pro-apoptotic serine/threonine kinases that have been implicated in regulation of apoptosis and tumour suppression. Expression of both Dlk/ZIP kinase and its interaction partner Par-4 is maintained in four medulloblastoma cell lines investigated, whereas three of seven neuroblastoma cell lines have lost expression of Par-4. Overexpression of a constitutively pro-apoptotic deletion mutant of Dlk/ZIP kinase induced significant apoptosis in D283 medulloblastoma cells. Cell death was characterized by apoptotic membrane blebbing, and a late stage during which the cells had ceased blebbing and were drastically shrunken or disrupted into apoptotic bodies. Over-expression of the anti-apoptotic Bcl-xL protein had no effect on Dlk/ZIP kinase-induced membrane blebbing, but potently inhibited Dlk/ZIP kinase-induced cytochrome c release and transition of cells to late stage apoptosis. Treatment with caspase inhibitors delayed, but did not prevent entry into late stage apoptosis. These results demonstrate that Dlk/ZIP kinase-triggered apoptosis involves the mitochondrial apoptosis pathway. However, cell death proceeded in the presence of caspase inhibitors, suggesting that Dlk/ZIP kinase is able to activate alternative cell death pathways. Alterations of signal transduction pathways leading to Dlk/ZIP kinase induced apoptosis or loss of expression of upstream activators could play important roles in tumour progression and metastasis of neural tumours. © 2001 Cancer Research Campaign http://www.bjcancer.com


*British Journal of Cancer* (2001) **85**(11), 1801–1808
© 2001 Cancer Research Campaign
doi: 10.1054/ bjoc.2001.2158, available online at http://www.idealibrary.com on IDEAL

### Dlk/ZIP kinase-induced apoptosis in human medulloblastoma cells: requirement of the mitochondrial apoptosis pathway

**D Kögel[1], C Reimertz[1], P Mech[1], M Poppe[1], MC Frühwald[3], H Engemann[4], KH Scheidtmann[4] and JHM Prehn[1,2]**

[1]Interdisciplinary Center for Clinical Research (IZKF), Research Group 'Apoptosis and Cell Death'; [2]Department of Pharmacology and Toxicology; [3]Department of Pediatric Hematology and Oncology, Faculty of Medicine, Westphalian Wilhelms-University, D-48149 Münster; and [4]Institute of Genetics, University of Bonn, D-53117 Bonn, Germany

**Summary** Dlk/ZIP kinase is a member of the Death Associated Protein (DAP) kinase family of pro-apoptotic serine/threonine kinases that have been implicated in regulation of apoptosis and tumour suppression. Expression of both Dlk/ZIP kinase and its interaction partner Par-4 is maintained in four medulloblastoma cell lines investigated, whereas three of seven neuroblastoma cell lines have lost expression of Par-4. Overexpression of a constitutively pro-apoptotic deletion mutant of Dlk/ZIP kinase induced significant apoptosis in D283 medulloblastoma cells. Cell death was characterized by apoptotic membrane blebbing, and a late stage during which the cells had ceased blebbing and were drastically shrunken or disrupted into apoptotic bodies. Over-expression of the anti-apoptotic Bcl-xL protein had no effect on Dlk/ZIP kinase-induced membrane blebbing, but potently inhibited Dlk/ZIP kinase-induced cytochrome c release and transition of cells to late stage apoptosis. Treatment with caspase inhibitors delayed, but did not prevent entry into late stage apoptosis. These results demonstrate that Dlk/ZIP kinase-triggered apoptosis involves the mitochondrial apoptosis pathway. However, cell death proceeded in the presence of caspase inhibitors, suggesting that Dlk/ZIP kinase is able to activate alternative cell death pathways. Alterations of signal transduction pathways leading to Dlk/ZIP kinase induced apoptosis or loss of expression of upstream activators could play important roles in tumour progression and metastasis of neural tumours. © 2001 Cancer Research Campaign http://www.bjcancer.com

**Keywords**: Dlk/ZIP kinase; DAP kinase family; apoptosis; mitochondria; Bcl-2 family; medulloblastoma

Apoptosis is an evolutionarily conserved form of cell suicide that functions to eliminate damaged and unnecessary cells. Key players involved in the apoptotic signalling network are the death receptors which directly activate the caspase cascade via apoptotic adaptor proteins (Ashkenazi and Dixit, 1998). In an alternative pathway, designated the mitochondrial pathway, translocation of proapoptotic bcl-2 family members (Bax, Bid) to the mitochondrial membrane and subsequent outer mitochondrial membrane permeabilization triggers release of apoptogenic factors such as cytochrome c, AIF (apoptosis-inducing factor) and Smac/DIABLO (Liu et al, 1996; Kroemer, 1999; Daugas et al, 2000; Du et al, 2000; Verhagen et al, 2000). In the presence of dATP, cytoplasmic cytochrome c forms a complex with APAF-1 and pro-caspase-9 (pro-Casp9), resulting in autocatalytic Casp9 activation (Li et al, 1997; Zou et al, 1997). Activated Casp9 then activates executioner caspase Casp3 (Li et al, 1997).

Recently, an increasing number of protein kinases has been shown to be involved in regulation of apoptosis (Cross et al, 2000). The DAP (death associated protein) kinase family is a group of pro-apoptotic serine/threonine kinases (Inbal et al, 2000; Kögel et al, 2001). The five members of this family are DAP kinase (Deiss et al, 1995), Dlk/ZIP kinase (Kögel et al, 1998, Kawai et al, 1998), DRP-1/DAPK2 (Kawai et al, 1999, Inbal et al, 2000), DRAK1 and DRAK2 (Sanjo et al, 1998). Dlk/ZIP kinase is expressed in the nervous system (Kögel et al, 1998). In non-apoptotic cells, Dlk/ZIP kinase is located in the nucleus where it interacts with transcription factors ATF4 and AATF (Kawai et al, 1998; Page et al, 1999a, Page et al, 1999b). The apoptotic activity of Dlk/ZIP kinase requires its relocation from the nucleus to the cytoplasm. One established model for relocation of Dlk/ZIP kinase involves complex formation of Dlk/ZIP kinase with the early apoptosis response factor Par-4 (Page et al, 1999b). However, the role of Dlk/ZIP kinase in tumour progression and metastasis and the apoptotic signalling pathways in which Dlk/ZIP kinase is embedded are not well understood to date. In the present study, we have determined the expression of Dlk/ZIP kinase and Par-4 in medulloblastoma and neuroblastoma cell lines. Additionally, we present evidence for the requirement of the mitochondrial pathway in Dlk/ZIP kinase-mediated apoptosis.

### MATERIALS AND METHODS

### Chemicals

Caspase-inhibitors Ac-Asp-Glu-Val-Asp-aldehyde (Ac-DEVD-CHO) and Ac-Tyr-Val-Ala-Asp-aldehyde (Ac-YVAD-CHO) were purchased from Bachem (Heidelberg, Germany), Z-Val-Ala-Asp(O-methyl)-fluoromethylketone (zVAD-fmk) was obtained from Enzyme Systems Products (Dublin, CA, USA).

### Semiquantitative RT-PCR

RT-PCR was performed with Ready to Go™ RT-PCR Beads (Amersham Pharmacia Biotech, Freiburg, Germany) according to

*Received 2 July 2001*
*Revised 5 September 2001*
*Accepted 17 September 2001*

*Correspondence to:* D Kögel the instructions of the manufacturer. Total RNA from human medulloblastoma cell lines Daoy, D341, MHH-MED-1 and D-283 and from human neuroblastoma cell lines IMR-5, SH-SY5Y, KCN, SHEP-SF, CH-LA-90, LA-N-6 and SK-M-FI was analysed for expression of Dlk/ZIP kinase and Par-4, with 18S-rRNA expression serving as an internal control. A 390 bp product of Dlk/ZIP kinase was amplified with primers 5′-CTACCTG-GTCAGCTGACCCAC-3′ and 5′-TGCTTGAGGAACTGGGT-GGC-3′ (30 PCR cycles), and a 501 bp product of Par-4 was amplified with primers 5′-AGAGACCTAGATGACATAGAAG-3′ and 5′-GGCACACATCAGTATAGGGAC-3′ (25 PCR cycles). The 397 bp 18S rRNA product was generated with primers 5′-TAGTTGGTGGAGCGATTTGTC-3′ and 5′-GGCCTCACTAAA CCATCCAAT-3′ (20 PCR cycles). All PCR reactions were run as follows: After the RT reaction the indicated number of PCR cycles (1 min denaturation at 95˚C, 1 min annealing at 56˚C and 1 min elongation at 72˚C) were performed.

### Cloning

Plasmids encoding GFP (green fluorescent protein) fusion proteins of wild type Dlk/ZIP kinase, kinase negative mutant K42A and deletion mutant ΔC2 have been described previously (Kögel et al, 1999). pGFP-Bax was generated by amplifying the complete ORF (codon 1–192) of human Bax-alpha with primers 5′-TTAGATC-TATGGACGGGTCCGGGGAG-3′ and 5′-AAGAATTCCCAT-CTTCTTCCAGATGGTGA-3′ using Pfu Polymerase (Promega, Mannheim, Germany). The obtained PCR product was then digested with *Bgl*II and *Eco*RI and cloned between the *Bgl*II and *Eco*RI sites of pGFP1-C1 (Clontech, Palo Alto, CA, USA).

### Cell culture and transfection

Generation of stable cell lines D-283 SFFV and D-283 bcl-xL which are derived from human D-283 medulloblastoma cells has been described previously (Poppe et al, 2001). Both cell lines were cultured in RPMI 1640 medium (Life Technologies, Germany) supplemented with penicillin (100 U/ml), streptomycin (100 U/ml) and 10% fetal calf serum (Life Technologies). For immunofluorescence microscopy experiments cells were grown on poly-L-lysine coated glass coverslips. For apoptosis assays cells were plated on 24-well tissue culture plates (Nunc, Hamburg, Germany) and transfected with a plasmid coding for GFP-ΔC2, a constitutively pro-apoptotic deletion mutant of Dlk/ZIP kinase (Kögel et al, 1999). 300 ng DNA and 2 μl Lipofectamine (Gibco BRL) were diluted in 300 μl RPMI medium under serum free conditions and incubated at room temperature for 30 min. The cultures were incubated with the DNA-Lipofectamine-transfection mixture at 37˚C and 5% $CO_2$ for 2 h. Cells were then cultured with RPMI medium containing 10% fetal calf serum. The apoptotic morphology of the cells was investigated by epifluorescence microscopy using an Eclipse TE 300 inverted microscope and a 20× objective (Nikon, Düsseldorf, Germany) equipped with the appropriate filter set (excitation of 465 to 495 nm, dichroic mirror of 505 nm and emission of 515 to 555 nm) for GFP fluorescence. For each transfection the apoptosis stage distribution of at least 100 GFP-positive cells was scored in triplicate. All experiments were repeated three times.

### Immunofluorescence analysis

For immunofluorescence analysis, cells were fixed on eight-well tissue culture slides by subsequent incubation with 2% paraformaldehyde in PBS at room temperature and pure methanol at –20˚C for 15 min each. After fixation the cells were washed three times with PBS, permeabilized at 4˚C for 3 min in PBS containing 0.1% Triton X-100 and then incubated with blocking solution (PBS with 5% horse serum and 0.3% Triton X-100) for 30 min at room temperature. Cytochrome c was detected using a monoclonal anti-cytochrome c antibody (clone 6H2.B4; Pharmingen Becton Dickinson) which recognizes the native form of cytochrome c. The antibody was used at a concentration of 1:1000 in PBS containing 1% horse serum and 0.3% Triton X-100. After incubation at room temperature for 2 h, cells were washed twice with PBS and incubated with biotin-conjugated anti-mouse IgG antibody (Vector Laboratories, Burlingame, CA) at a dilution of 1:500. The second antibody was detected using Texas-Red conjugated streptavidin (Molecular Probes) diluted 1:1000 in PBS for 20 min at room temperature. Epifluorescence microscopy was observed with the Eclipse TE 300 inverted microscope and a 40 × oil immersion objective. Texas Red fluorescence was observed with the following optics: excitation 540–580 nm; dichroic mirror, 595 nm; emission, 600 to 660 nm. Digital images were acquired with a SPOT-2 camera using Spot software version 2.2.1 (Diagnostic Instruments, Sterling Heights, MI).

### RESULTS

### Expression analysis of Dlk/ZIP kinase and Par-4 in human medulloblastoma and neuroblastoma cell lines

Dlk/ZIP kinase expression has been demonstrated in various tissues, including mammalian brain (Kögel et al, 1999). The expression of Dlk/ZIP kinase was investigated by RT-PCR analysis of neuronal tumour cell lines. Dlk/ZIP kinase was expressed in all five medulloblastoma cell lines and in all seven neuroblastoma cell

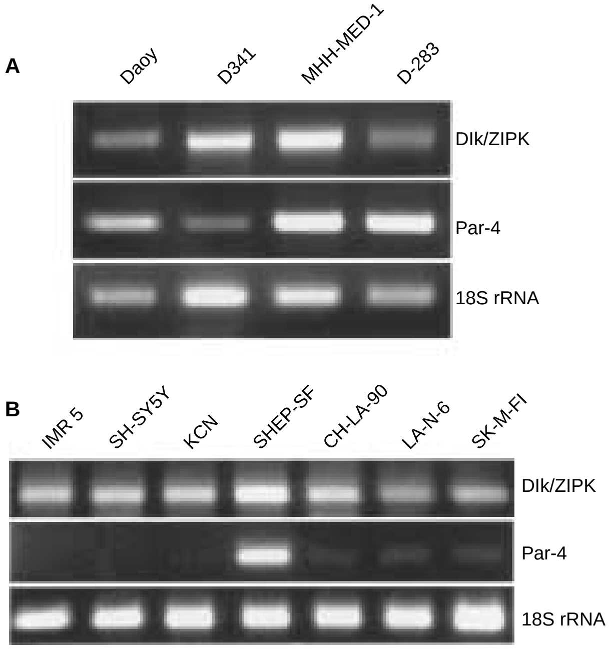

**Figure 1** Expression of both Dlk/ZIP kinase and Par-4 in human medulloblastoma and neuroblastoma cell lines as analysed by RT-PCR. cDNA from cultured cell lines Daoy, D341, MHH-MED-1, D-283, IMR-5, SH-SY5Y, KCN, SHEP-SF, CH-LA-90, LA-N-6 and SK-M-FI was amplified by 20 cycles (18S rRNA), 25 cycles (Par-4) or 30 cycles (Dlk/ZIP kinase) of PCR. Reaction products were separated by agarose gel electrophoresis and visualized with 0.1% ethidium bromide

 *© 2001 Cancer Research Campaign*

lines tested (Figure 1A). Interestingly, significant basal expression of the Dlk/ZIP kinase interaction partner Par-4 is observed in only one of the neuroblastoma cell lines, SHEP-SF (Figure 1B), with three of the other cell lines, IMR-5, SH-SY5Y and KCN having completely lost expression. In contrast, all five medulloblastoma cell lines retain expression of Par-4 (Figure 1A) and also DAP (death associated protein) kinase (data not shown).

### Transient over-expression of a constitutively pro-apoptotic mutant of Dlk/ZIP kinase induces apoptosis in D-283 medulloblastoma cells

Dlk/ZIP kinase is normally localized in the nucleus where it partially associates with PML (promyelocytic leukaemia) bodies (Kögel et al, 1999). Transient overexpression of wild type Dlk/ZIP kinase fused to GFP in D-283 cells revealed the typical speckled nuclear fluorescence, while a kinase negative mutant, GFP-K42A was diffusely distributed in the nucleus (Figure 2) as observed previously in rat fibroblasts (Kögel et al, 1999). Wild type GFP-Dlk/ZIP kinase alone does not induce apoptosis to a significant extent. However, coexpression of Dlk/ZIP kinase and Par-4 leads to cytoplasmic retention of Dlk/ZIP kinase and apoptosis (Page et al, 1999b). In addition, a deletion mutant, GFP-ΔC2 which is lacking the functional NLS of Dlk/ZIP kinase and consequently is located in the cytoplasm induces apoptosis with high efficiency (Kögel et al, 1999). GFP-ΔC2 does not require complex formation with Par-4 for induction of apoptosis but otherwise mimicks all events that occur in Dlk-Par-4-mediated apoptosis (Kögel et al, 1999; Page et al, 1999a). Therefore, GFP-ΔC2 represents a constitutively pro-apoptotic mutant of Dlk/ZIP kinase. Expression of GFP-ΔC2 in D-283 cells potently induced apoptosis (Figure 2). Based on morphological criteria, apoptosis triggered by Dlk/ZIP kinase was divided into

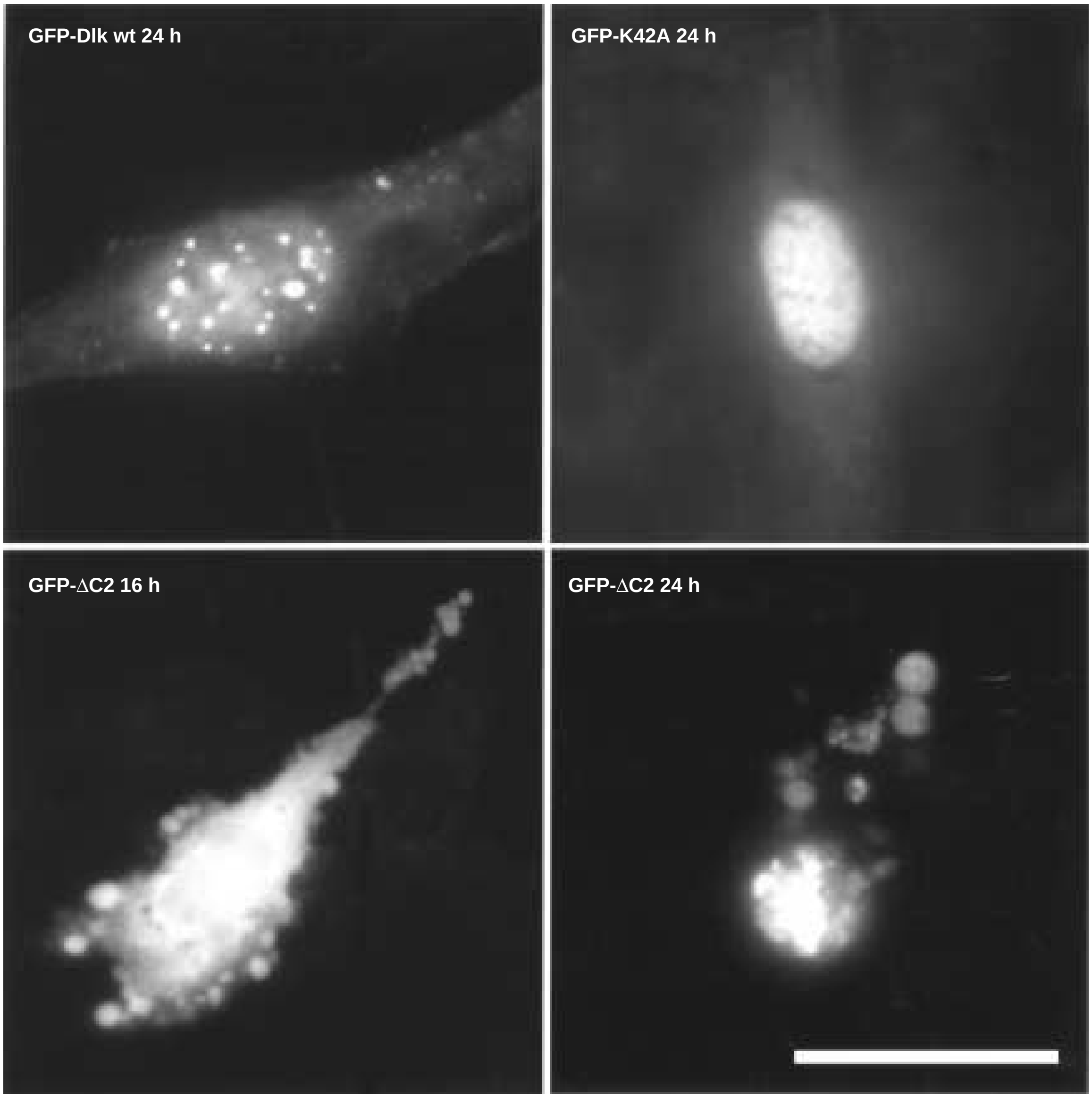

**Figure 2** Dlk/ZIP kinase-induced apoptosis in human D-283 medulloblastoma cells can be divided into distinct stages. D-283 SFFV cells were transiently transfected with plasmids encoding GFP fusion proteins of Dlk/ZIP kinase. Wild type Dlk/ZIP kinase (GFP-Dlk-wt) and kinase negative mutant K42A (GFP-K42A) are localized in the nucleus and do not trigger apoptosis. Constitutively pro-apotpotic mutant GFP-ΔC2 induces an early membrane blebbing stage and a late apoptotic stage (right panel). Live cells were analyzed by epifluorescence microscopy after 16 and 24 h and digital images were acquired. Scale bar = 25 µm

© 2001 Cancer Research Campaign three distinct stages: in stage I, the cells reveal no morphological signs of apoptosis, stage II is characterized by extensive membrane blebbing without significant reduction of cell volume, while in stage III the cells have ceased blebbing, were drastically shrunken, detached or even disrupted into apoptotic bodies.

### Bcl-xL inhibits cytochrome c release and late stage Dlk/ZIP kinase-mediated apoptosis, but does not abrogate Dlk-induced membrane blebbing

To analyse the involvement of mitochondrial events in Dlk/ZIP kinase-mediated apoptosis we employed cell lines D-283 SFFV and D-283 bcl-xL the latter stably over-expressing the antiapoptotic bcl-2 family member Bcl-xL (Poppe et al, 2001). Although overexpression of GFP-ΔC2 in D-283 bcl-xL cells also lead to extensive membrane blebbing, the majority of cells did not proceed to the late stage of Dlk/ZIP kinase-mediated apoptosis (Figure 3A, right panels). Figures 3B and C show the quantification of apoptosis stage distribution from three independent experiments (per treatment) after 24 h and 42 h, respectively. Late stage apoptosis was observed in 51 +/– 4% (after 24 h) and 91+/– 2% (after 42 h) of D-283 SFFV cells expressing GFP-ΔC2. In contrast, overexpression of Bcl-xL led to late stage apoptosis in only 9 +/– 3% (after 24 h) and 28 +/– 12% (after 42 h) of GFP-ΔC2-expressing cells. Thus, Bcl-xL overexpression arrests the cells in the membrane blebbing stage by inhibiting the transition from the early stage to the late stage of Dlk/ZIP kinase-mediated apotosis.

The fact that Bcl-xL overexpression inhibited late stage Dlk/ZIP kinase-mediated apoptosis implied that a mitochondrial step played a crucial role downstream of Dlk/ZIP kinase activity. Indeed, Dlk/ZIP kinase-mediated apoptosis triggered by mutant GFP-ΔC2 was associated with release of cytochrome c from the mitochondria as indicated by a diffuse cytoplasmic and nuclear staining in late stage apoptotic D-283 SFFV cells (Figures 4A and B). A similar immunofluorescence pattern was observed in cells transfected with GFP-Bax. Over-expression of GFP-Bax was sufficent to induce apoptosis without any additional apoptotic stimulus and the GFP-Bax signal was clustered in the perinuclear area indicative of mitochondrial localization (Figures 4E and F). Bcl-xL overexpression inhibited cytochrome c release induced by GFP-ΔC2 (Figure 4G and H). In cells transfected with GFP-Bax, overexpression of Bcl-xL blocked cytochrome c release as well as translocation of GFP-Bax to the mitochondria, as indicated by the diffuse distribution of the GFP-Bax fluorescence (Figures 4I and J).

### Dlk/ZIP kinase-mediated apotosis proceeds in the presence of caspase inhibition

Addition of the broad range caspase inhibitor zVAD-fmk also delayed apoptosis triggered by GFP-ΔC2 in D-283-SFFV cells, but to a much lesser extent than Bcl-xL overexpression. 41 +/– 7 and 63 +/– 10% of GFP-positive cells were in the late stage after 24 h and 42 h, respectively (Figures 3B and C). Treatment of D-283 bcl-xL cells with zVAD-fmk did not lead to a synergistic protection against Dlk/ZIP kinase mediated apoptosis. The apoptosis stage distribution of D-283 SFFV cells was also quantified 42 h after transfection in the presence of caspase inhibitors Ac-DEVD-CHO and Ac-YVAD-CHO (Figure 5). Treatment with Ac-DEVD-CHO which inhibits Casp3, Casp7 and Casp8, but also Casp1, Casp6, Casp9 and Casp10 decreased the amount of cells in the late stage of apoptosis from 87 +/– 4% (control, without inhibitor) to 70 +/– 8%. Finally, treatment with Ac-YVAD-CHO which inhibits Casp1, Casp4 and Casp5 had no effect at all on apoptosis stage distribution (85 +/– 7% of cells in the late stage). However, we did not observe disruption of cells into apoptotic bodies in the presence of either zVAD-fmk (Figure 2A, middle panels) or Ac-DEVD-CHO (data not shown). Taken together, these results suggest that caspase activation is not required for entry into stage III but for late executionary processes during Dlk/ZIP kinase-mediated apoptosis.

### DISCUSSION

The present study demonstrates that Dlk/ZIP kinase expression is retained in medulloblastoma and neuroblastoma cell lines. The proapoptotic activity of Dlk/ZIP kinase has been suggested to require complex formation with the early apoptosis response factor Par-4 and retargeting of this complex to the cytoskeleton (Page et al, 1999a, b). There is emerging evidence that Par-4 is critically involved in neuronal apoptosis (Mattson et al, 1999). Interestingly, expression of Par-4 is lost in three of seven neuroblastoma cell lines investigated, IMR-5, SH-SY5Y and KCN (Figure 1). Par-4 upregulation is a very early event during apoptosis and precedes caspase activation and mitochondrial dysfunction (Guo et al, 1999). In addition to activation of Dlk/ZIP kinase, Par-4 promotes apoptosis by inhibiting the antiapoptotic NF-κB pathway. This is achieved by binding of Par-4 to another protein kinase, the NF-κB upstream activator PKCζ (Berra et al, 1997; Diaz-Meco et al, 1999; Camandola and Mattson, 2000). Thus, Par-4 may represent a central coordinator during the early stages of apoptosis, which is capable of integrating different pro-(Dlk/ZIP kinase) and anti-apoptotic (NF-κB) signalling pathways by complexing individual components of these pathways.

Dlk/ZIP kinase-induced cell death involves the activation of the evolutionarily conserved apoptotic cell death machinery, comprising cytochrome c release, caspase activation, as well as cell death regulation by anti-apoptotic Bcl-2 family proteins. Over-expression of Bcl-xL efficiently blocked cytochrome c release and late stage apoptosis. In addition, over-expression of Bcl-2 has been shown to rescue cell death induced by activated DAP kinase (Cohen et al, 1999). Pro-apoptotic signalling of DAP kinase family members may generally require the activation of the mitochondrial apoptosis pathway. In contrast to Bcl-xL over-expression, caspase inhibition just delayed the progression of cells from the early to the late stage of Dlk/ZIP kinase-mediated apoptosis. This suggests that Dlk/ZIP kinase also induced Bcl-xL-sensitive, but caspase-independent cell death pathways. Indeed, it is well-established that programmed cell death can occur in the absence of caspase activity (Borner and Monney, 1999). Antiapoptotic bcl-2 family members are capable of blocking several cell death pathways induced by selective mitochondrial outer membrane permeabilization during apoptosis. Following permeabilization of the mitochondrial membrane, a variety of proapoptotic factors are released that could trigger apoptosis. Nuclear chromatin condensation induced by the mitochondrial release of AIF has been shown to occur in a caspase-independent fashion (Daugas et al, 2000). Furthermore, release of cytochrome c may lead to an increased generation of reactive oxygen species

 *© 2001 Cancer Research Campaign*

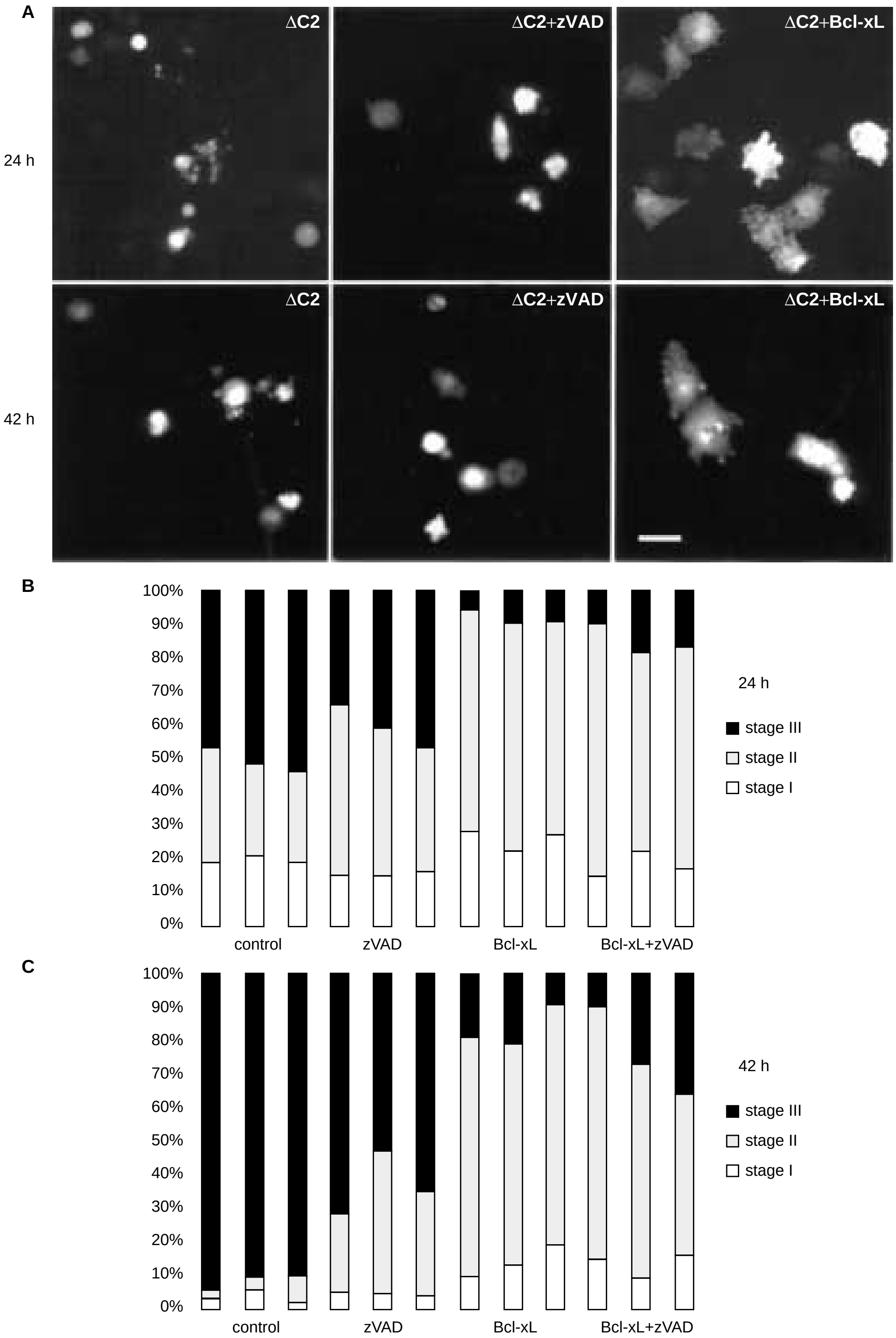

**Figure 3** **A** Late stage apoptosis induced by DIk/ZIP kinase is inhibited by Bcl-xL. Deletion mutant GFP-ΔC2 was transiently transfected into cell lines D-283 SFFV (stably transfected with parental vector pSFFV, left and middle panels) and D-283 bcl-xL (stably over-expressing Bcl-xL, right panels) by lipofection. Where indicated, broad range caspase inhibitor zVAD-fmk was added to a final concentration of 100 μM immediately after removal of the transfection supernatant. Live cells were analysed by epifluorescence microscopy after 24 and 42 h and digital images were acquired. Scale bar = 25 μm. **B** and **C** Quantitative analysis of apoptosis stage distribution in cell lines D-283 SFFV and D-283 bcl-xL transfected with DIk/ZIP kinase deletion mutant GFP-ΔC2. Each column represents one individual experiment. After 24 (**B**) and 42 (**C**) h, 100 GFP-positive cells per single experiment were counted for apoptotic morphology using epifluorescence microscopy. Where indicated, broad range caspase inhibitor zVAD-fmk was added to a final concentration of 100 μM. Experiments were repeated three times with comparable results

*© 2001 Cancer Research Campaign* 

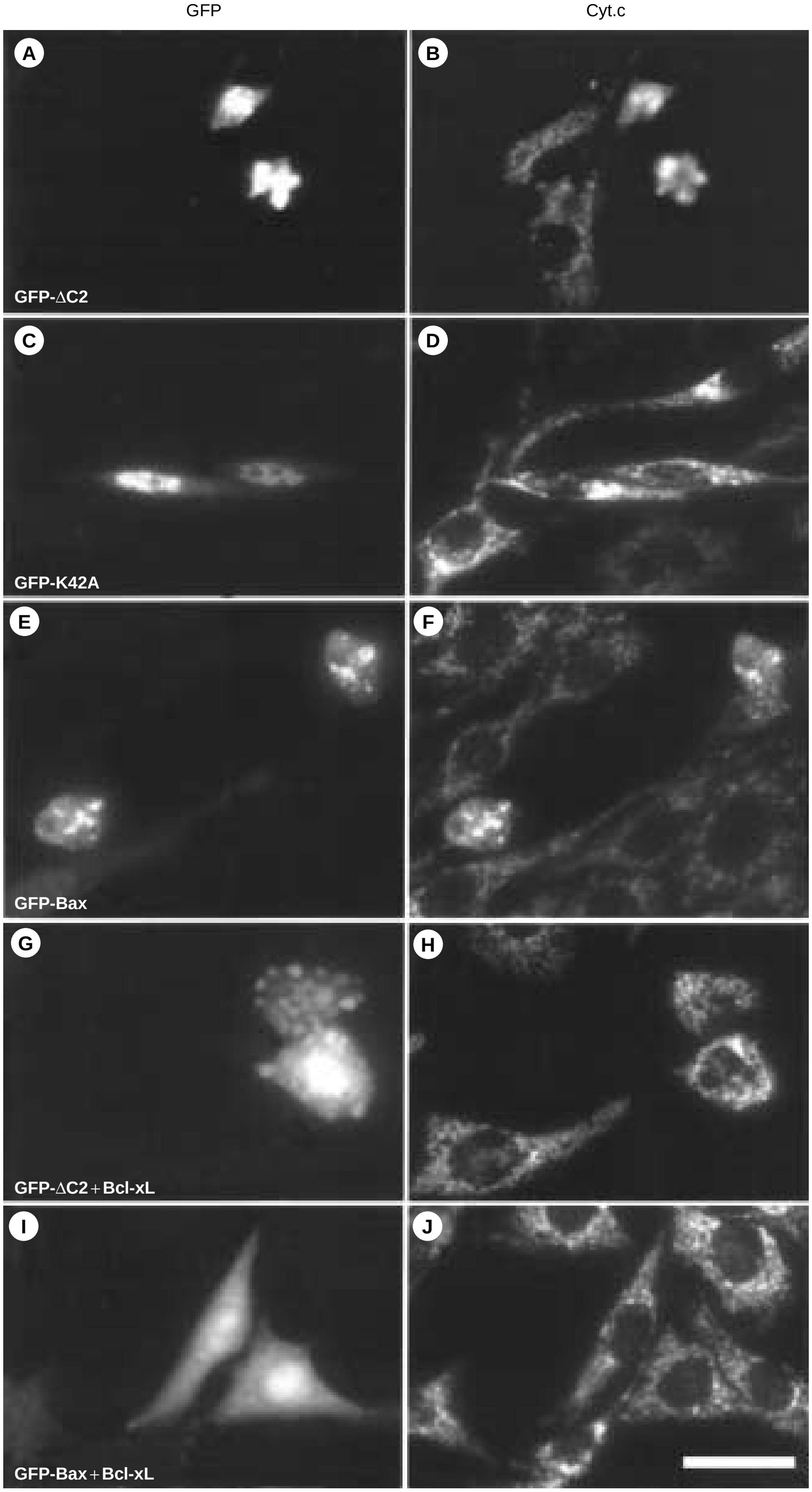

**Figure 4** Late stage Dlk/ZIP kinase-mediated apoptosis is associated with release of cytochrome c from the mitochondria. Cell lines D-283 SFFV (**A** to **F**) and D-283 bcl-xL (**G** to **J**) were transienty transfected with the indicated GFP-constructs and the cells were fixed after 24 h. Subcellular distribution of cytochrome c was investigated by immunofluorescence analysis and images of representative cells were acquired. Scale bar = 25 μm

 *© 2001 Cancer Research Campaign*

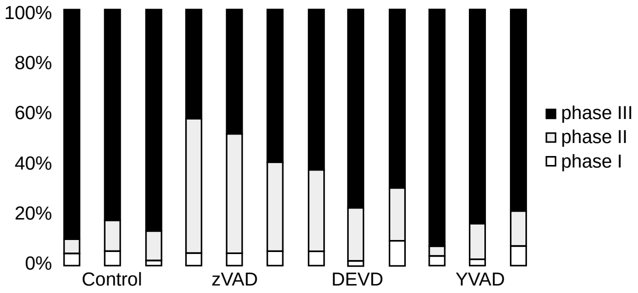

**Figure 5** Dlk/ZIP kinase-mediated apoptosis is associated with caspase 3-like activity. Quantitative analysis of apoptosis stage distribution in cell line D-283 SFFV transfected with Dlk/ZIP kinase deletion mutant GFP-ΔC2. After 42 h, 100 GFP-positive cells per single experiment were counted for apoptotic morphology using epifluorescence microscopy. Caspase inhibitors Ac-DEVD-CHO and Ac-YVAD-CHO were added to a final concentration of 10 μM, broad range caspase inhibitor zVAD-fmk to a final concentration of 100 μM. Experiments were repeated three times with comparable results

(ROS) during apoptosis (Loeffler and Kroemer, 2000; Luetjens et al, 2000). It is conceivable that generation of ROS and release of AIF from the mitochondria are involved in apoptosis triggered by DAP kinase family members. The nuclear apoptosis regulator PML has also been implicated in caspase-independent cell death pathways (Quignon et al, 1998; Guo et al, 2000). Interestingly, nuclear Dlk/ZIP kinase colocalizes with PML bodies (Kögel et al, 1999), but the significance of this association is unclear at present.

Even though Bcl-xL over-expression inhibited late stage apoptosis induced by Dlk/ZIP kinase, it had no effect on Dlk/ZIP kinase-triggered membrane blebbing. Protection by Bcl-xL thus occured downstream or independent of the initial membrane blebbing step. Hence, Dlk/ZIP kinase likely activates alternative biochemical pathways responsible for morphological changes during apoptosis. Dlk/ZIP kinase induces significant membrane blebbing in cells (Kögel et al, 1999). Membrane blebbing is considered a hallmark of extranuclear apoptosis and an early event in apoptotic cell death (Mills et al, 1999). Two recent reports identified ROCK I as a caspase-dependent effector of apoptotic membrane blebbing (Coleman et al, 2001, Sebbagh et al, 2001). However, membrane blebbing has been shown to be caspase-independent in several apoptotic systems (McCarthy et al, 1999; Mills et al, 1998). Likewise, membrane blebbing induced by Dlk/ZIP kinase was neither inhibited by Bcl-xL, nor by the broad spectrum caspase inhibitor zVAD-fmk. Myosin light chain (MLC) phosphorylation has been implicated as a critical event in apoptotic membrane blebbing (Mills et al, 1998; Coleman et al, 2001; Sebbagh et al, 2001). Interestingly, DAP kinase family members share high sequence homology with MLC kinases and several members, including Dlk/ZIP kinase have been shown to phosphorylate MLC in vitro (Kögel et al, 1998; Murata-Hori et al, 1999). Thus, DAP kinase family members are likely candidates for cytoplasmic effectors of apoptotic membrane blebbing.

Loss of DAP kinase expression has been shown in a variety of human tumours (reviewed in Cohen and Kimchi, 2001). In addition, DAP kinase family members might be inactivated on the posttranslational level, either by absence of coactivators such as Par-4 or by anti-apoptotic antagonizers such as Bcl-xL. Interestingly, basal expression of Par-4 was lost in three of seven neuroblastoma cell lines investigated. Inactivation of DAP kinase family members might be critically involved in tumorigenesis and metastasis of neural tumors.

### ACKNOWLEDGEMENTS

We thank Christiane Schettler for technical assistance. These experiments were supported by grants from the Deutsche Forschungsgemeinschaft (Pr 338/9–1) and IZKF Universität Münster (BMBF 01 KS 9604/0) to JHMP

### REFERENCES

Ashkenazi A and Dixit VM (1998) Death receptors: signaling and modulation. *Science* **281**: 1305–1308

Berra E, Municio MM, Sanz L, Frutos S, Diaz-Meco MT and Moscat J (1997) Positioning atypical protein kinase C isoforms in the UV-induced apoptotic signaling cascade. *Mol Cell Biol* **17**: 4346–4354

Borner C and Monney L (1999) Apoptosis without caspases: an inefficient molecular guillotine? *Cell Death Differ* **6**: 497–507

Camandola S and Mattson MP (2000) Pro-apoptotic action of PAR-4 involves inhibition of NF-kappaB activity and suppression of BCL-2 expression. *J Neurosci Res* **61**: 134–139

Cecconi F (1999) Apaf1 and the apoptotic machinery. *Cell Death Differ* **6**: 1087–1098

Cohen O, Feinstein E and Kimchi A (1997) DAP-kinase is a Ca2+/calmodulin-dependent, cytoskeletal-associated protein kinase, with cell death-inducing functions that depend on its catalytic activity. *Embo J* **16**: 998–1008

Cohen O, Inbal B, Kissil JL, Raveh T, Berissi H, Spivak-Kroizaman T, Feinstein E and Kimchi A (1999) DAP-kinase participates in TNF-alpha- and Fas-induced apoptosis and its function requires the death domain. *J Cell Biol* **146**: 141–148

Cohen O and Kimchi A (2001) DAP-kinase: from functional gene cloning to establishment of its role in apoptosis and cancer. *Cell Death Differ* **8**: 6–15

Coleman ML, Sahai EA, Yeo M, Bosch M, Dewar A and Olson MF (2001) Membrane blebbing during apoptosis results from caspase-mediated activation of ROCK 1. *Nat Cell Biol* **3**: 339–345

Cross TG, Scheel-Toellner D, Henriquez NV, Deacon E, Salmon M and Lord JM (2000) Serine/threonine protein kinases and apoptosis. *Exp Cell Res* **256**: 34–41

Daugas E, Susin SA, Zamzami N, Ferri KF, Irinopoulou T, Larochette N, Prevost MC, Leber B, Andrews D, Penninger J and Kroemer G (2000) Mitochondrio-nuclear translocation of AIF in apoptosis and necrosis. *FASEB J* **14**: 729–739

Deiss LP, Feinstein E, Berissi H, Cohen O and Kimchi A (1995) Identification of a novel serine/threonine kinase and a novel 15-kD protein as potential mediators of the gamma interferon-induced cell death. *Genes Dev* **9**: 15–30

Desagher S and Martinou JC (2000) Mitochondria as the central control point of apoptosis. *Trends Cell Biol* **10**: 369–377

Diaz-Meco MT, Lallena MJ, Monjas A, Frutos S and Moscat J (1999) Inactivation of the inhibitory kappaB protein kinase/nuclear factor kappaB pathway by Par-4 expression potentiates tumor necrosis factor alpha-induced apoptosis. *J Biol Chem* **274**: 19606–19612

Du C, Fang M, Li Y, Li L and Wang X (2000) Smac, a mitochondrial protein that promotes cytochrome c-dependent caspase activation by eliminating IAP inhibition. *Cell* **102**: 33–42

Guo Q, Fu W, Xie J, Luo H, Sells SF, Geddes JW, Bondada V, Rangnekar VM and Mattson MP (1999) Par-4 is a mediator of neuronal degeneration associated with the pathogenesis of Alzheimer disease. *Nat Med* **4**: 957–962

Guo A, Salomoni P, Luo J, Shih A, Zhong S, Gu W and Paolo Pandolfi P (2000) The function of PML in p53-dependent apoptosis. *Nat Cell Biol* **2**: 730–736

Inbal B, Cohen O, Polak-Charcon S, Kopolovic J, Vadai E, Eisenbach L and Kimchi A (1997) DAP kinase links the control of apoptosis to metastasis. *Nature* **390**: 180–184

Inbal B, Shani G, Cohen O, Kissil JL and Kimchi A (2000) Death-associated protein kinase-related protein 1, a novel serine/threonine kinase involved in apoptosis. *Mol Cell Biol* **20**: 1044–1054

Kawai T, Matsumoto M, Takeda K, Sanjo H and Akira S (1998) ZIP kinase, a novel serine/threonine kinase which mediates apoptosis. *Mol Cell Biol* **18**: 1642–1651

Kawai T, Nomura F, Hoshino K, Copeland NG, Gilbert DJ, Jenkins NA and Akira S (1999) Death-associated protein kinase 2 is a new calcium/calmodulin-

© 2001 Cancer Research Campaign 

dependent protein kinase that signals apoptosis through its catalytic activity. *Oncogene* **18**: 3471–3480

Kissil JL, Feinstein E, Cohen O, Jones PA, Tsai YC, Knowles MA, Eydmann ME and Kimchi A (1997) DAP-kinase loss of expression in various carcinoma and B-cell lymphoma cell lines: possible implications for role as tumor suppressor gene. *Oncogene* **15**: 403–407

Kögel D, Plöttner O, Landsberg G, Christian S and Scheidtmann KH (1998) Cloning and characterization of DIk, a novel serine/threonine kinase that is tightly associated with chromatin and phosphorylates core histones. *Oncogene* **17**: 2645–2654

Kögel D, Bierbaum H, Preuss U and Scheidtmann KH (1999) C-terminal truncation of DIk/ZIP kinase leads to abrogation of nuclear transport and high apoptotic activity. *Oncogene* **18**: 7212–7218

Kögel D, Prehn JH and Scheidtmann KH (2001) The DAP kinase family of pro-apoptotic proteins: novel players in the apoptotic game. *Bioessays* **23**: 352–358

Kroemer G (1999) Cytochrome c. *Curr Biol* **9**: R468

Li P, Nijhawan D, Budihardjo I, Srinivasula SM, Ahmad M, Alnemri ES and Wang X (1997) Cytochrome c and dATP-dependent formation of Apaf-1/caspase-9 complex initiates an apoptotic protease cascade. *Cell* **91**: 479–489

Liu X, Kim CN, Yang J, Jemmerson R and Wang X (1996) Induction of apoptotic program in cell-free extracts: requirement for dATP and cytochrome c. *Cell* **86**: 147–157

Loeffler M and Kroemer G (2000) The mitochondrion in cell death control: certainties and incognita. *Exp Cell Res* **256**: 19–26

Luetjens CM, Bui NT, Sengpiel B, Munstermann G, Poppe M, Krohn AJ, Bauerbach E, Krieglstein J and Prehn JH (2000) Delayed mitochondrial dysfunction in excitotoxic neuron death: cytochrome c release and a secondary increase in superoxide production. *J Neurosci* **20**: 5715–5723

Mattson MP, Duan W, Chan SL and Camandola S (1999) Par-4: an emerging pivotal player in neuronal apoptosis and neurodegenerative disorders. *J Mol Neurosci* **13**: 17–30

McCarthy NJ, Whyte MK, Gilbert CS and Evan GI (1999) Inhibition of Ced-3/ICE-related proteases does not prevent cell death induced by oncogenes, DNA damage, or the Bcl-2 homologue Bak. *J Cell Biol* **136**: 215–227

Mills JC, Stone NL, Erhardt J and Pittman RN (1998) Apoptotic membrane blebbing is regulated by myosin light chain phosphorylation. *J Cell Biol* **140**: 627–636

Mills JC, Stone NL and Pittman RN (1999) Extranuclear apoptosis. The role of the cytoplasm in the execution phase. *J Cell Biol* **146**: 703–708

Murata-Hori M, Suizu F, Iwasaki T, Kikuchi A and Hosoya H (1999) ZIP kinase identified as a novel myosin regulatory light chain kinase in HeLa cells. *FEBS Lett* **451**: 81–84

Page G, Kögel D, Rangnekar V and Scheidtmann KH (1999a) Interaction partners of DIk/ZIP kinase: co-expression of DIk/ZIP kinase and Par-4 results in cytoplasmic retention and apoptosis. *Oncogene* **18**: 7265–7273

Page G, Lödige I, Kögel D and Scheidtmann KH (1999b) AATF, a novel transcription factor that interacts with DIk/ZIP kinase and interferes with apoptosis. *FEBS Lett* **462**: 187–191

Poppe M, Reimertz C, Krohn AJ, Luetjens CM, Böckelmann D, Nieminen A-L, Kögel D and Prehn JHM (2001) Dissipation of mitochondrial potassium and proton gradients inhibits cytochrome c release during neuronal apoptosis. *J Neurosci* **21**: 4551–4563

Quignon F, De Bels F, Koken M, Feunteun J, Ameisen JC and de The H (1998) PML induces a novel caspase-independent death process. *Nat Genet* **20**: 259–265

Sanjo H, Kawai T and Akira S (1998) DRAKs, novel serine/threonine kinases related to death-associated protein kinase that trigger apoptosis. *J Biol Chem* **273**: 29066–29071

Sebbagh M, Renvoize C, Hamelin J, Riche N, Bertoglio J and Breard J (2001) Caspase-3-mediated cleavage of ROCK I induces MLC phosphorylation and apoptotic membrane blebbing. *Nat Cell Biol* **3**: 346–352

Verhagen AM, Ekert PG, Pakusch M, Silke J, Connolly LM, Reid GE, Moritz RL, Simpson RJ and Vaux DL (2000) Identification of DIABLO, a mammalian protein that promotes apoptosis by binding to and antagonizing IAP proteins. *Cell* **102**: 43–53

Zou H, Henzel WJ, Liu X, Lutschg A and Wang X (1997) Apaf-1, a human protein homologous to C. elegans CED-4, participates in cytochrome c-dependent activation of caspase-3. *Cell* **90**: 405–413

*© 2001 Cancer Research Campaign*
